# Direct application of an ECG-based sleep staging algorithm on reflective photoplethysmography data decreases performance

**DOI:** 10.1186/s13104-020-05355-0

**Published:** 2020-11-10

**Authors:** M. M. van Gilst, B. M. Wulterkens, P. Fonseca, M. Radha, M. Ross, A. Moreau, A. Cerny, P. Anderer, X. Long, J. P. van Dijk, S. Overeem

**Affiliations:** 1 Department of Electrical Engineering, Eindhoven University of Technology, PO Box 513, 5600 MB Eindhoven, The Netherlands; 2 Philips Research, High Tech Campus 34, 5656 AE Eindhoven, The Netherlands; 3 Sleep and Respiratory Care, Home Healthcare Solutions, Philips Austria GmbH, Kranichberggasse 4, 1120 Vienna, Austria; 4 Sleep Medicine Centre Kempenhaeghe, Sterkselseweg 65, 5591 VE Heeze, The Netherlands

**Keywords:** Heart rate variability, Sleep staging, Wearable, Polysomnography, Validation, Sleep disorders

## Abstract

**Objective:**

The maturation of neural network-based techniques in combination with the availability of large sleep datasets has increased the interest in alternative methods of sleep monitoring. For unobtrusive sleep staging, the most promising algorithms are based on heart rate variability computed from inter-beat intervals (IBIs) derived from ECG-data. The practical application of these algorithms is even more promising when alternative ways of obtaining IBIs, such as wrist-worn photoplethysmography (PPG) can be used. However, studies validating sleep staging algorithms directly on PPG-based data are limited.

**Results:**

We applied an automatic sleep staging algorithm trained and validated on ECG-data directly on inter-beat intervals derived from a wrist-worn PPG sensor, in 389 polysomnographic recordings of patients with a variety of sleep disorders. While the algorithm reached moderate agreement with gold standard polysomnography, the performance was significantly lower when applied on PPG- versus ECG-derived heart rate variability data (kappa 0.56 versus 0.60, p < 0.001; accuracy 73.0% versus 75.9% p < 0.001). These results show that direct application of an algorithm on a different source of data may negatively affect performance. Algorithms need to be validated using each data source and re-training should be considered whenever possible.

## Introduction

Polysomnography (PSG) remains the gold standard for objective sleep monitoring, despite obvious disadvantages such as obtrusiveness, costs associated with data acquisition and analysis, and unsuitability for long-term recordings. Because of these limitations, alternative methods to record sleep and associated pathological events gain increasing interest. Gaining insight in the sleep structure, the cyclicity of sleep stages, is a key element in the diagnosis of sleep disorders. A promising example of a surrogate sleep staging technique is the use of cardiorespiratory measures, most notably heart rate variability (HRV). HRV-based algorithms that allow sleep–wake detection, but also three- or four-class sleep stage classifiers reached promising performance compared to PSG [1–5]. While the concept of HRV-based sleep staging has been recognized for quite some time, the approach is gaining increased attention, due to innovations in neural network-based techniques combined with the availability of large sleep datasets.

Most well-validated HRV-algorithms are developed on inter-beat intervals (IBIs) derived from ECG data. One obvious advantage of HRV-based methods is the potential to apply the algorithms on IBI-measurements obtained by non-obtrusive alternatives for ECG, such as reflective photoplethysmography (PPG) in wrist-worn devices. This technique is widely used in consumer devices, intended to gain insight in physical activity, energy expenditure and sleep. However, in many of these devices it is impossible to access the raw PPG data, which limits current applicability in clinical and research settings [6].

Retraining an HRV-model on PPG data requires large prospective studies, whereas ECG data can be obtained retrospectively from clinical PSGs routinely performed in sleep centers. To our knowledge, only two studies have been published that incorporate raw PPG signals for the development of automatic sleep staging algorithms. Both studies were performed in healthy participants [1, 7]. While it is tempting to consider HRV-based methods as ‘sensor agnostic’, the performance effects of direct application of ECG-based algorithms to PPG-derived data should be specifically studied.

We recently described an HRV-based automatic sleep staging algorithm, trained and validated on ECG data from a broad cohort of unselected sleep disordered patients [3]. Here, we apply this algorithm to IBIs obtained by a wrist-worn PPG sensor, to assess performance of the algorithm on raw PPG data and investigate the effect of direct application of a machine learning approach on a different type of raw data without re-training.

## Methods

### Algorithm and dataset

Previously, we developed a machine learning approach for automatic ECG-based sleep staging with ECG-derived HRV, based on long short-term memory (LSTM) recurrent neural networks [8]. We retrained the algorithm on a separate dataset including healthy sleepers and sleep disordered patients, and validated it on an independent broad cohort of unselected sleep disordered patients [3]. Here, we directly apply the ECG-based algorithm on HRV-data obtained by wrist-worn PPG in the same validation set.

Data was derived from the Sleep and OSA Monitoring with Non-Invasive Applications (SOMNIA) database containing a prospective cohort of patients with various sleep disorders from a tertiary sleep center [9]. The study was approved by the medical ethical committee of the Maxima Medical Center (Veldhoven, The Netherlands, N16.074), and all participants provided written informed consent. Here, we used the first 389 recordings which included PSG and time-synchronized data from a wrist-worn sensor measuring reflective PPG and accelerometry (Royal Philips, Amsterdam, The Netherlands) [9].

Patient demographics are listed in Table 1. Sleep stages were scored in 30s epochs according to the 2015 AASM criteria [10]. The resulting ground-truth reference classes were obtained by combining N1 and N2 in a single “N1/N2” class while the remaining classes (Wake, N3 and REM) were used without changes. For details on sleep staging and clinical diagnosis of the patients, see Fonseca et al. [3].

**Table 1:** Patient demographics

Parameter	
N	389
N Female (%)	145 (37.3%)
Age (years)	51.1 ± 14.8
BMI (kg/m^2^)	27.7 ± 5.0
*Primary sleep diagnostic category*^a^	Total prevalence (%)
Sleep disordered breathing	224 (57.6)
Insomnia	110 (28.3)
Movement disorder	48 (12.3)
Behavioral	33 (8.5)
REM parasomnia	19 (4.9)
Non-REM parasomnia	13 (3.3)
Other	23 (5.9)

^a^Patients could be diagnosed with more than one sleep disorder

To compute the HRV features as described in previous research [3, 8], individual heartbeats were first detected from the raw PPG signal using a template-based beat segmentation algorithm [11]. The time difference between each pair of heartbeats was calculated and implausible IBIs with a duration lower than 0.3 s or higher than 1.5 s were excluded. Gross body movements were quantified as activity counts for each 30 s of the recording based on the three-axial accelerometer signal (see [3]).

### Performance measures and statistics

Sleep staging performance using PPG data was compared to gold standard PSG using measures previously described [3, 8]. In short, epoch-per-epoch agreement between the predicted classes and PSG sleep stages was assessed using two quality metrics: accuracy and Cohen’s kappa coefficient of agreement (or κ). Agreement was computed for four classes, three classes (merging N1/N2 and N3 in a single non-REM “NREM” class), and two classes (merging all sleep stages in a single “Sleep” class). For the latter, we calculated sensitivity, specificity and positive predictive value (PPV), all in respect to the detection of the positive class, i.e. Wake. To test the algorithm’s capacity to detect specific sleep stages, a similar analysis was performed for the remaining classes (N1/N2, N3, and REM), considering each class in comparison with the merged remaining classes.

The effect of demographic characteristics on four-class performance was examined using the Wilcoxon rank-sum test to assess influence of sex, and Spearman’s correlations to evaluate effects of age and BMI.

The performance of the algorithm using PPG data was compared to the performance of the algorithm using the ECG signal, as originally presented in [3]. We used the same participants in both studies, enabling us to make a paired performance comparison. A Wilcoxon signed-rank test was applied to compare both kappa and accuracy from both four-class sleep staging results. Furthermore, we compared the coverage of the ECG and PPG signal, defined as the percentage of the recording where we could detect valid IBIs from the signals of each sensor. Spearman’s correlation was used to assess whether the difference in coverage could explain the difference in performance between ECG and PPG data input. Differences in performance were also evaluated with respect to age and sex using Spearman’s correlation and Wilcoxon signed-rank tests respectively.

All data are represented as mean ± SD unless otherwise stated.

## Results

### Sleep staging performance

Table 2 shows the agreement for each classification task, between the predicted sleep stages and the sleep stages classified using PSG. The classifier performs the best for the REM class, with an average κ of 0.64 and a sensitivity of 79.8%. The worst performing class is N3, with an average κ of 0.51 and an average sensitivity of 50.7%. Two-class (wake/sleep) sleep stage prediction shows an average κ of 0.57, a sensitivity of 67.8% and a specificity of 91.9%. Significant (p < 0.001) but weak Spearman’s rank correlation coefficients were found between age and κ (ρ = − 0.25), BMI and κ (ρ = − 0.12) and age and accuracy (ρ = − 0.21).

**Table 2:** Epoch-per-epoch agreement between predicted sleep stages based on PPG and ground-truth for different classification tasks

Task	kappa (–)	Accuracy (%)	Sensitivity (%)	Specificity (%)	PPV (%)
Wake/N1–2/N3/REM	0.56 ± 0.15	73.0 ± 9.4	n/a	n/a	n/a
Wake/NREM/REM	0.62 ± 0.16	81.4 ± 8.5	n/a	n/a	n/a
Wake/sleep^a^	0.57 ± 0.18	87.7 ± 8.1	67.8 ± 19.9	91.9 ± 8.4	68.4 ± 19.6
N1–2^a^	0.49 ± 0.16	75.1 ± 8.3	77.1 ± 10.9	72.6 ± 13.6	75.9 ± 12.3
N3^a^	0.51 ± 0.24	91.2 ± 5.2	50.7 ± 26.4	97.6 ± 3.2	75.5 ± 26.6
REM^a^	0.64 ± 0.22	91.9 ± 4.5	79.8 ± 21.8	93.6 ± 4.1	64.6 ± 21.0

*PPV* positive predictive value

^a^Binary classification tasks were evaluated in a one vs. rest strategy, where one single class (wake, N1–N2, or N3, or REM) was considered the ‘positive’ class, and the remaining classes were merged in a single ‘negative’ class. All results are presented as mean ± SD

### Performance comparison PPG- versus ECG-based HRV

The algorithm performed worse when using PPG-derived versus ECG-derived IBIs. There was a significant difference in four-class sleep staging performance between the PPG- and ECG-based results on both kappa (PPG κ = 0.56 ± 0.15; ECG κ = 0.60 ± 0.14; p < 0.001) and accuracy (PPG 73.0 ± 9.4%; ECG 75.9 ± 8.5%; p < 0.001). The correlation between performance difference (ECG-PPG) to the difference in signal coverage throughout the night showed a small but significant correlation with both kappa (ρ = 0.25, p < 0.001) and accuracy (ρ = 0.25, p < 0.001). No significant correlations were found between the differences in performance and age or sex. The drop in performance was similar across all sleep disorders.

## Discussion

Recently, we developed, trained and validated a sleep staging algorithm based on HRV derived from ECG data [3, 8]. In the current study, we applied this algorithm directly, without re-training, to IBIs derived from raw PPG in 389 subjects with varying sleep disorders. Overall, the classifier achieved moderate agreement with gold standard PSG, with an average κ of 0.56 and accuracy of 73.0%. However, performance of the algorithm on PPG-data was significantly lower than using ECG. This indicates that a direct application of HRV-based sleep staging algorithms on a different source of measurement data is not trivial and may hamper reliability.

Several mechanisms may lead to changes in performance when using PPG- instead of ECG-derived IBIs. Performance differences correlated with a difference in coverage of detectable IBIs between ECG and PPG throughout the night, although the explained variance was very low (r^2^ = 0.063). In our assessment of signal coverage, we only checked whether IBIs were physiologically plausible, but not whether they actually correspond to the actual distance between consecutive heart beats. For example, under certain conditions such as during periodic limb movements, and given the susceptibility of this sensor modality to motion artifacts, the signal morphology might resemble pulse amplitude changes typical of heart beats thus yielding invalid IBIs. In such situations, the difference between ECG and PPG might be even larger.

In general, the PPG signal is more susceptible to motion artifacts because of larger movements in the extremities (as compared to the thorax) and worse coupling between the sensor and the skin. This can further impair the extraction of HRV features [12]. Motion artifacts may be present to a varying degree depending on sleep stage and thus differentially affect staging performance compared to ECG-based data. The pressure between the photosensor and the skin can also affect PPG signal quality. For example, too little pressure can lead to displacement of the sensor. On the other hand, too much pressure between the photosensor and skin (e.g. when lying on the sensor) can cause increased constriction of the arterioles perfusing the skin. As a consequence, both signal amplitude and signal-to-noise ratio decrease, complicating accurate localization of individual heartbeats [13]. Artifacts can also be a result of large changes in venous blood due to limb movements, especially in case of low perfusion at the sensor site. The pulsatile components in the signal are then composed of more than just arterial blood, leading to a false derivation of heartbeats [14].

Several other mechanisms may contribute to differences in beat-to-beat intervals measured with ECG and from the pulse-wave signal. Pulse transit time (PTT), the time for the pulse pressure wave to travel between the heart to the peripheral circulation, may be affected by blood pressure [15]. Blood pressure may vary differently across sleep stages and by influencing PTT thus have an effect on PPG-derived beat intervals. Sleep-stage dependent variations in peripheral artery constriction and dilation may differently affect pulse wave velocity [16].

Our data supports the notion that HRV-based sleep staging is a promising tool with various advantages, most notably the ability to do long-term monitoring of sleep in an unobtrusive way. However, the measurement principle is not completely sensor-agnostic and performance can be influenced by the measurement modality. Most large datasets comprising gold standard PSG only contain ECG as a means to obtain HRV, so it is likely that the best performing algorithms will be developed on this data source. However, it is not sufficient to directly apply ECG-based algorithms to other modalities such as wrist-worn PPG. At the least, performance needs to be validated by comparison with the gold standard. Moreover, re-training of the algorithm on the specific data source should be considered whenever possible. Alternatively, or in addition, methods for domain adaptation such as teacher-student paradigms [17] or transfer learning [18, 19] could be used to increase performance for the new sensor. To do so, there is a need for large prospective datasets containing new methods of acquiring physiological data in combination with polysomnography, not only in healthy subjects but in clinical populations as well.

### Limitations

In this study we evaluated only one sleep staging algorithm. For other algorithms the difference between ECG- and PPG-based scoring might be smaller. However, as shown in the discussion, there are several physiological aspects to be taken into account when detecting HRV features from different data sources. Therefore algorithms should always be re-validated when using a new sensor modality.

## Data Availability

The SOMNIA data used in this study are available from the Sleep Medicine Centre Kempenhaeghe upon reasonable request. Specific restrictions apply to the availability of the data collected with sensors not comprised in the standard PSG set-up (i.e. PPG data), since these sensors are used under license and are not publicly available. These data are however available from the authors upon reasonable request and with permission of the licensors. The algorithm used in this study is extensively described in previous publications [3, 8].This manuscript focusses on general methodological questions, therefore the code is not published.

## Abbreviations

BMI: Body mass index
ECG: Electrocardiography
EEG: Electroencephalography
HRV: Heart rate variability
IBI: Inter beat interval
NREM: Non-REM, non-rapid eye movement (sleep)
OSA: Obstructive sleep apnea
PPG: Photoplethysmography
PSG: Polysomnography
PTT: Pulse transit time
REM: Rapid eye movement (sleep)
